# Buoyancy-Free Janus Microcylinders as Mobile Microelectrode Arrays for Continuous Microfluidic Biomolecule Collection within a Wide Frequency Range: A Numerical Simulation Study

**DOI:** 10.3390/mi11030289

**Published:** 2020-03-10

**Authors:** Weiyu Liu, Yukun Ren, Ye Tao, Hui Yan, Congda Xiao, Qisheng Wu

**Affiliations:** 1 School of Electronics and Control Engineering, Chang’an University, Middle-Section of Nan’er Huan Road, Xi’an 710064, China; liuweiyu@chd.edu.cn (W.L.); 2016904117@chd.edu.cn (C.X.); qshwu@chd.edu.cn (Q.W.); 2 State Key Laboratory of Robotics and System, Harbin Institute of Technology, West Da-zhi Street 92, Harbin 150001, China; tarahit@gmail.com; 3 School of Mechatronics Engineering, Harbin Institute of Technology, West Da-zhi Street 92, Harbin 150001, China

**Keywords:** induced-charge electrokinetic phenomenon, ego-dielectrophoresis, mobile electrode, Janus microsphere, continuous biomolecule collection, electroconvection

## Abstract

We numerically study herein the AC electrokinetic motion of Janus mobile microelectrode (ME) arrays in electrolyte solution in a wide field frequency, which holds great potential for biomedical applications. A fully coupled physical model, which incorporates the fluid-structure interaction under the synergy of induced-charge electroosmotic (ICEO) slipping and interfacial Maxwell stress, is developed for this purpose. A freely suspended Janus cylinder free from buoyancy, whose main body is made of polystyrene, while half of the particle surface is coated with a thin conducting film of negligible thickness, will react actively on application of an AC signal. In the low-frequency limit, induced-charge electrophoretic (ICEP) translation occurs due to symmetric breaking in ICEO slipping, which renders the insulating end to move ahead. At higher field frequencies, a brand-new electrokinetic transport phenomenon called “ego-dielectrophoresis (e-DEP)” arises due to the action of the localized uneven field on the inhomogeneous particle dipole moment. In stark contrast with the low-frequency ICEP translation, the high-frequency e-DEP force tends to drive the asymmetric dipole moment to move in the direction of the conducting end. The bidirectional transport feature of Janus microspheres in a wide AC frequency range can be vividly interpreted as an array of ME for continuous loading of secondary bioparticles from the surrounding liquid medium along its direction-controllable path by long-range electroconvection. These results pave the way for achieving flexible and high-throughput on-chip extraction of nanoscale biological contents for subsequent on-site bioassay based upon AC electrokinetics of Janus ME arrays.

## 1. Introduction

Lab-on-a-chip technology requires the development of new methods to manipulate small fluid and particle entities at micrometer dimension [1,2]. Discrete electrode array embedded in microfabricated fluidic networks stands for a brand new hope for direct electrokinetic actuation either on liquid suspension [3,4,5,6] or solid particles [7,8,9] dispersed in the fluid. Electrokinetics (EK) and electrohydrodynamics (EHD) of leaky dielectric medium [10,11] in microsystems have received unprecedentedly increasing attention from the microfluidic society for the last two decades. Traditional linear electroosmosis (EO) [12], electrowetting on dielectrics [13], induction EHD [14], injection EHD [15], conduction EHD [16], as well as nonlinear electroosmosis [17] are all typical representatives of physical phenomena where an external electric field is applied for driving fluids on nanoliter scale.

The common trait of EK and EHD is characterized by an active interaction between local electric field and the space charge cloud induced by itself to exert net electrostatic body forces that drive the motion of liquid medium, suspending colloids, discrete droplets as well as biological content of the microfluidic system, in the context of the so-called Ohm model [18,19,20] in close connection with electrochemical polarization [21,22,23,24] at a charged solid/electrolyte interface. The fast advance of microfabrication technique during the last ten years has allowed for an ease with which conducting metal plates can be patterned and inserted into microfluidic devices. For such, DC and AC electric fields as well as their delicate combinations have been widely employed for manipulating particle and liquid contents of microsystems [25,26].

In terms of the physical origin of the relevant charge layers, the primary electrical force exerting on microscale solid entities suspended in aqueous electrolytes includes electrophoresis (EP) [27,28,29], nonlinear induced-charge electrophoresis (ICEP) [30,31,32,33,34,35], and dielectrophoresis [36,37,38]. Directed “force-free” electrophoretic delivery of microspheres in a constant DC electric field is caused by the linear electroosmosis fluid stress on the surface of the natively charged particles, which is due to the interaction between an imposed tangential field and the natural diffuse screening cloud adjacent to the target sample suspended in aqueous electrolyte [39].

Unlike EP, both ICEP and dielectrophoresis (DEP) depend quadratically on the applied voltage, so they are nonlinear electrokinetic effects, wherein an applied electric field acts on its own induced charge to engender even time-averaged electrostatic particle motion under AC forcing. In ICEP, the induce charge cloud inside the electric double layer (EDL) is due to electrochemical polarization of ideally polarizable surfaces under the influence of an external electric field [34]. Different from ICEP, DEP arises from the bipolar surface charge, both free and bound, induced at the particle/solution interface via the mechanism of Maxwell-Wagner structural polarization [40,41].

In general, for symmetric particle entities, DEP and ICEP can only occur in a field gradient, so that symmetry breaking in electrical polarization induces a net electrokinetic motion along or against the gradient of electric field strength [10]. In fact, DEP and ICEP always tend to counterbalance one another, and gold spheres should keep stationary in DC limits from a theoretical perspective [42]. Nevertheless, dipolophoresis takes place in static fields in practical experiments; in that the present theory of ICEP would routinely overestimate the ICEO flow velocity by about one to two orders of magnitude, resulting in the dominating role of DEP over ICEP [43,44].

The intricate interplay between DEP and ICEP in dipolophoresis of symmetric metal colloids in field gradients makes dipolophoresis of ideally polarizable particles quite impractical for application in real experiments. So, great attention has been paid to the ICEP translation of Janus colloids during the last decade [33,45,46,47,48]. A Janus particle, whose half body is far less polarizable and another half is far more polarizable than the liquid suspension, serves as a typical example of solid entity with natural symmetry breaking in geometry and/or electrical properties [49]. In this way, ICEP translation of Janus colloids readily occurs in a uniform electric field even in the absence of a field gradient [50], which renders them good candidates for serving as mobile electrodes. Of course, such conducting electrodes embedded in microfluidic channels can also be fabricated using alternative materials such as liquid metal, as has been well studied by pioneering researchers [51,52,53,54,55,56].

**Figure 1:**
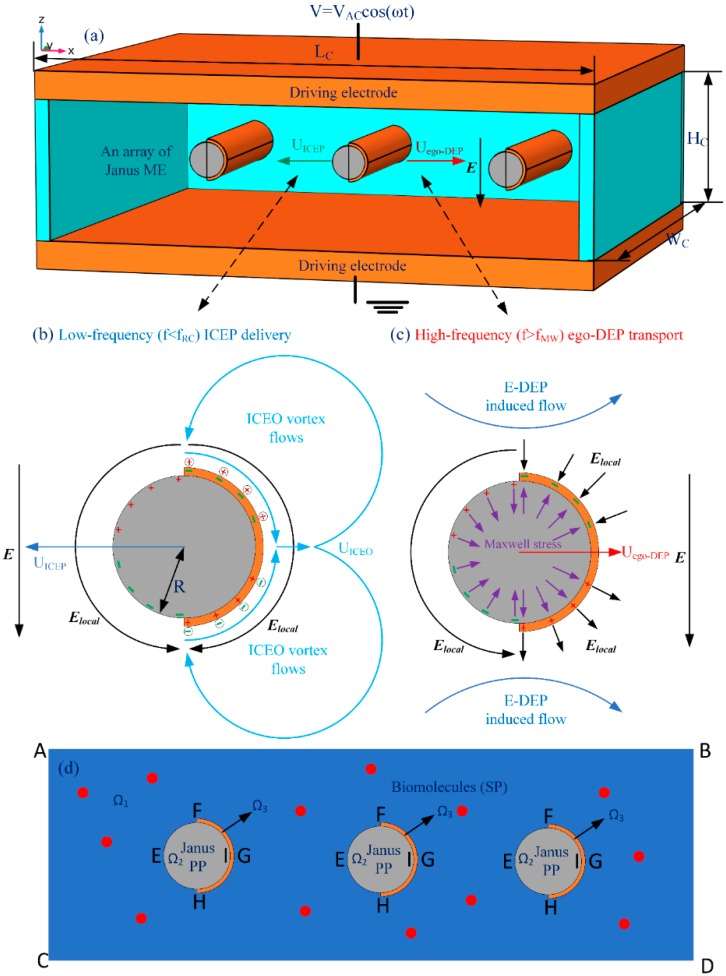
A schematic diagram of the direction-controllable electrokinetic transport of an array of cylindrical Janus mobile microelectrodes (ME) freely suspended in aqueous electrolyte under a uniform AC electric field, with the specific geometric size given in Table 1. (**a**) A 3D schematic diagram of the buoyancy-free Janus ME array that can move unidirectionally in perpendicular orientation to the externally-imposed AC forcing within a wide frequency range. (**b**) At frequencies below the inverse RC time scale for electrochemical polarization on the ideally polarizable surface of thin gold membrane, the induced-charge electrophoretic (ICEP) effect associated with induced-charge electroosmotic (ICEO) vortex flow field on the conducing end propels electrohydrodynamically the Janus ME to move in the direction of the insulating end. (**c**) At frequencies approaching and even exceeding the reciprocal charge relaxation time of the bulk electrolyte, ICEO vanishes due to electrochemical ion relaxation, and a net Maxwell stress from Maxwell-Wagner interfacial polarization renders the Janus ME array move electrokinetically in the direction of the conducting end, namely, the phenomenon of “ego-dielectrophoresis (e-DEP)”. (**d**) An illustrative simulation domain for the electrokinetic motion of buoyancy-free Janus microspheres (primary particle, PP) as mobile microelectrode arrays for microfluidic biomolecule (secondary particle, SP) collection within a wide frequency range. It is noteworthy that the background AC electric field is uniform and there is no field gradient far from the Janus ME array.

It has been reported that, for a Janus microsphere subjected to a uniform low-frequency AC forcing, symmetry breaking in ICEO would first induce a net rotating motion due to combined ICEP stress and electro-orientational (ER) torque (See Supporting Video S1). A steady state is reached when the hemisphere interface aligns with the field axis, with the specific azimuth angle determined by its initial orientation. Then, the strong ICEO vortex flow field around the conducting end would push the Janus particle to transport unidirectionally with its insulating end moving ahead in the low-frequency limit, as shown in Figure 1, which is perpendicular to the AC forcing.

The research group of Yossifon has recently discovered that a Janus colloid can also move perpendicular to the applied field for frequency even higher than the inverse RC time constant for electrochemical polarization of the conducting side, a phenomenon termed “self-dielectrophoresis (s-DEP)” [57]. More importantly, the potential use of such active particles as carriers of both organic and inorganic biological cargo was recently discovered and reported by the same great group as well [58,59]. In s-DEP, an asymmetric microsphere always sediments to the channel floor due to a large mass density of the conducting hemisphere in comparison to that of the liquid suspension. So, a strong electric field intensity is produced inside the narrow gap between the bottom section of the particle and the channel floor. This localized field gradient interacts actively with the asymmetric induced dipole moment, and thereby produces a net time-averaged electrostatic force that tends to transport the Janus colloid with its conducting side moving ahead [58], resulting in a reversal in the translating motion due to s-DEP with respect to that driven by ICEP. For s-DEP to occur, nevertheless, besides sinking to the channel bottom surface, the field frequency is strictly confined to be no more than the reciprocal Debye relaxation time of the liquid bulk.

To address the above issue, we reinvestigated the frequency-dependence of the translating behavior of Janus colloids in a unique configuration of microfluidic device, wherein a pair of face-to-face oppositely polarized parallel-plate electrode sandwiches a central microscale fluidic channel of a rectangular cross section (Figure 1a). To keep the cost of the memory within our available computer resource, a 2D calculation domain representing the x-z cross section was considered for the numerical simulation, wherein a Janus cylinder or an array of Janus mobile microelectrodes (ME) is suspended in a conducting buffer medium with its long axis coinciding with the y axis. Different from previous cases, the solid Janus entity in current study has a uniform body made of polystyrene (PS), and the surface of its half body is coated with a thin film of ideally polarizable noble metal material (such as gold). So, in the present case, it can be assumed that the Janus ME is freely suspended in water electrolyte, and thereby no sedimentation occurs.

In essence, the phenomenon of s-DEP would not happen without a close contact of the asymmetric particle dipole moment with the insulating substrate. In the current situation, however, the Janus entity is neutral in buoyancy effect, and thereby it is not possible for the physical condition of s-DEP-induced translation to be satisfied. In fact, up to now, to the best of our knowledge, all the literatures regarding electrokinetics of Janus particles (JP) focused on the motion of JPs near the wall or finally move close to the channel bottom surface. That is, there is still no such experimental data about the electrokinetic behavior of JP far away from the wall. In order to confirm whether or not a directed motion akin to s-DEP can still occur for a freely suspended Janus entity, its electrokinetic behavior as a function of time is numerically calculated by using a fully coupled mathematical model with a transient solver. In the current model, both the effects that induced-charge electroosmotic (ICEO) flow and Maxwell-Wager interfacial polarization have on the fluid-structure interaction are taken into account, and we run the simulation in a broad frequency range from DC limit to beyond the charge relaxation time of the liquid bulk. A brand new electrokinetic translating behavior, called “ego-DEP (e-DEP)” is found in the high-frequency limit. Unlike s-DEP, e-DEP requires neither a close contact between the particle and substrate surface, nor a limit of high-frequency range to be no more than the inverse Debye relaxation time. The discovery of the phenomenon of e-DEP enriches the electrodynamic behavior of buoyancy-free asymmetric Janus mobile microelectrodes (ME), which holds great potential in on-chip biomedical applications.

## 2. Methods

### 2.1. Device Configuration

The geometry of the microfluidic device is shown Figure 1a. The cubic fluidic chamber has a length of *L_C_*, a width of *W_C_*, and a height of *H_C_*, with the specific channel dimensions shown in Table 1. The top and bottom walls of the channel consist of a pair of parallel conducting plates (indicated by the orange color), while all other sidewalls are frequency-independent ideal insulators in comparison with the leaky dielectric saline solution.

A microscale Janus cylinder or an array of such Janus mobile microelectrodes (ME), with the long axis orienting perfectly along the y dimension, is freely suspended in the central level of the thin liquid layer. Half of its PS body is in direct contact with the electrolyte, while another half has a thin coating layer of ideally polarizable gold material of a small but finite thickness of *H_mem_* = 50 nm that inhibits sedimentation. The polar interface bisecting the asymmetric cylindrical entity has a specific azimuth angle *θ* with respect to the horizontal x-axis. This kind of Janus cylinder can be chemically synthesized by bulk phase process on an industrial scale as compared to those methods based on the use of interfaces to break the symmetry [60]. It is found that the half PS body with thin coatings of gold membrane is an almost equal-potential half body under external AC forcing due to an induction process akin to electrostatic screening (not shown). So, e-DEP is able to still occur for a complete more polarizable hemisphere. However, e-DEP requires the JP to be freely suspended in the electrolyte solution without sedimentation, so that we prefer to make use of the thin coating case throughout this work.

On application of a standing AC voltage signal *V* = V_AC_·cos(*ωt*), a possibly inclined Janus cylinder will react by combined ICEP and e-DEP. It will first realign its central polar interface in the direction of the electric field by a torque of an electric origin, then transport unidirectionally with its insulating or conducting end moving ahead, as depending on whether the applied field frequency is in the low (ICEP) or high (e-DEP) frequency range. The bi-directional electrokinetic behavior can be exploited for continuous loading and collection of secondary biomolecules as the array of Janus ME transports unidirectionally within the channel (Figure 1d).

### 2.2. Mathematical Model

#### 2.2.1. AC Electric Field

The whole computational domain can be divided into four interrelated subregions, including the electrolyte bulk, the PS body of the Janus colloid, the thin gold-film coating, as well as the induced double layer (IDL) formed at the interface between the conducting film and liquid medium. The former three are treated as bulk zones, while the latter is represented by a conjugating condition of a sharp voltage drop across the IDL according to charge conservation equation. In the quasi-electrostatic limit, the electric potential field $\tilde{\phi }$ in all the three bulk regions is governed by the current continuity condition in the sinusoidal steady state [61,62,63]:

(1) $$\nabla \cdot \left(\left(\sigma_{i}+j\omega \varepsilon_{i}\right)\nabla {\tilde{\phi }}_{i}\right)=0\;\mathrm{for}\;i\;=\;1,\;2,\;3\;$$

where the subscripts *i* = 1, 2, 3 represent the electrolyte, PS material, and the gold membrane in sequence (Figure 1d). *σ* and *ε* denote the electric conductivity and dielectric permittivity of the corresponding material. With uniform electrical properties for each domain, the control equations of the electric fields are reduced to the Laplace equations with zero induced charge [64,65,66]:

(2) $$\nabla^{2}{\tilde{\phi }}_{i}=0\;\mathrm{for}\;i\;=\;1,\;2,\;3$$

At the interface between the liquid medium and the bare PS body, we have the following conjugating conditions [67]:

(3) $$\begin{array}{c} \left(\sigma_{1}+j\omega \varepsilon_{1}\right)\nabla {\tilde{\phi }}_{1}\cdot n=j\omega \varepsilon_{2}\nabla {\tilde{\phi }}_{2}\cdot n\\ {\tilde{\phi }}_{1}\;=\;{\tilde{\phi }}_{2} \end{array}$$

where we have assumed zero bulk conductivity of the PS main body. The polarizability of the PS material is too low to induce observable electrochemical polarization at the PS/medium interface. At the PS/gold interface, similar conjugating conditions are applied:

(4) $$\begin{array}{c} j\omega \varepsilon_{2}\nabla {\tilde{\phi }}_{2}\cdot n=\sigma_{3}\nabla {\tilde{\phi }}_{3}\cdot n\\ {\tilde{\phi }}_{2}\;=\;{\tilde{\phi }}_{3} \end{array}$$

At the membrane/electrolyte interface, the voltage drop across the induced double layer is related to the normal electric field right outside the Debye layer according to electric current continuity [68,69,70,71,72,73,74]:

(5) $$\begin{array}{c} \left(\sigma_{1}+j\omega \varepsilon_{1}\right)\nabla {\tilde{\phi }}_{1}\cdot n=j\omega C_{0}\left({\tilde{\phi }}_{1}-{\tilde{\phi }}_{3}\right)\\ \sigma_{3}\nabla {\tilde{\phi }}_{3}\cdot n=j\omega C_{0}\left({\tilde{\phi }}_{3}-{\tilde{\phi }}_{1}\right) \end{array}$$

where we have invoked the classical linear RC-circuit theory of field-induced Debye screening, under the limit of small double-layer voltage drop and thin boundary layer. *C*_0_ = *C_D_*/(1 + *δ*) is the total IDL capacitance, which is in effect a series connection of the diffuse layer capacity *C_D_* = *ε*_1_/*λ_D_* and the Stern layer capacity *C_S_*, in terms of the Debye screening length *λ_D_* = (*Dε*_1_/*σ*_1_)^1/2^ and the surface capacitance ratio of *δ* = *C_D_*/*C_S_*.

Only a part of the total double-layer voltage drops across the diffuse layer of mobile ions, namely, the induced zeta potential $\tilde{\zeta }={\tilde{\phi }}_{3}-{\tilde{\phi }}_{1}/1+\delta$. This contributes to a sharp velocity discontinuity at the membrane surface due to the action of induced-charge electroosmotic flow [75,76,77]:

(6) 〈uslip〉=−ε12ηRe(ζ˜E˜t*)=ε12η(1+δ)Re((ϕ˜1−ϕ˜3)(E˜1−E˜1⋅n⋅n)*)

where *η* = 0.001 Pa·s denotes the dynamic viscosity of the aqueous suspension.

On the conducting surface of a pair of face-to-face electrode plates, double-layer polarization is omitted considering the specific working frequency band, so fixed AC voltage phasor is imposed to establish a time-harmonic voltage difference along the channel height direction (Figure 1a):

(7) $${\tilde{\phi }}_{1}=V_{1}\;\mathrm{at}\;\mathrm{the}\;\mathrm{top}\;\mathrm{electrode}\;\mathrm{plate}\;{\tilde{\phi }}_{1}\;=\;0\;\;\mathrm{at}\;\mathrm{the}\;\mathrm{bottom}\;\mathrm{counterpart}$$

At the two end boundaries of the computational domain, the normal component of the total electric current vanishes to mimic a finite calculation volume:

(8) $$\nabla {\tilde{\phi }}_{1}\cdot n=0$$

#### 2.2.2. Particle Motion

The Janus entity is treated as linear elastic material; its displacement ***S*** is governed by its solid stress tensor ${\overset{↔}{T}}_{S}\left(S\right)$:

(9) $$\nabla \cdot {\overset{↔}{T}}_{S}\left(S\right)\;+\;f=\;0$$

where ***f*** is a source term of body force density acting on the asymmetric particle. In current analysis, we exclude the existence of any kind of body force by imposing ***f*** = 0, since the particle is freely suspended in the electrolyte solution with an almost neutral mass density. The Cauchy stress of the solid phase ${\overset{↔}{T}}_{S}\left(S\right)$ is clearly a function of the displacement S of the Janus ME.

Considering that the Janus entities are incompressible neo-Hookean material, which can be described by the strain energy density function, as given by:

(10) $$W=G_{0}\left(I_{C}-3\right)/2$$

where *G*_0_ is the shear modulus of the intrinsically deformable particles, *I_C_* = tr(**C**) denotes the first invariant of the right Cauchy–Green tensor. C(E,ν) = **F**_T_**F**, with *E* the Young modulus, ν the Poisson’s ratio, **F** the deformation gradient tensor that can be computed by **F** = ▽**S** + **I**.

The corresponding Cauchy stress of the neo-Hookean material can be described by:

(11) $${\overset{↔}{T}}_{S}\left(S\right){\;=\;J}^{-1}PF^{T}$$

where J is the determinant of the deformation gradient tensor **F**, **J** = 1 for an incompressible Neo-Hookean material, and **P** = *∂Ws*/∂∇*_X_**S*** is the first Piola–Kirchhoff stress.

Since the Janus ME used in current analysis is rigid and non-deformable, we arbitrarily set the Young modulus as large as 10^12^ Pa, while the Poisson’s ratio has a normal value of 0.33 to approach a rigid body. In this way, our model can be easily extended to describe the electrokinetic motion of deformable Janus cylinders with different shear modulus. That is, we make use of the model for deformable particles to equivalently describe the fluid-structure interactions of rigid Janus ME arrays by setting an extremely high Young modulus, which is feasible according to the correct simulation results in the section of numerical validation (Section 3.1).

#### 2.2.3. Fluid Motion

We solve the Navier–Stokes equation for incompressible Newtonian fluid to acquire the transient flow field of the liquid suspension:

(12) $$\rho \frac{\partial u_{f}}{\partial t}+\rho \left(u_{f}\cdot \nabla \right)u_{f}=\nabla \cdot \left(-p\overset{↔}{I}+\eta \left(\nabla u_{f}+\nabla u_{f}^{T}\right)\right)\;\;\;\nabla \cdot u_{f}=0$$

where *ρ* denotes the liquid mass density, p the hydraulic pressure, and ***u**_f_* the flow velocity field.

#### 2.2.4. Electric-Field-Mediated Particle-Fluid Interaction

At the particle/fluid interface, the physical constraint of total stress balance implies:

(13) $${\overset{↔}{T}}_{S}\left(S\right)\cdot n+{\overset{↔}{T}}_{H}\cdot n+\langle {\overset{↔}{T}}_{E}\rangle \cdot n=0$$

where ${\overset{↔}{T}}_{S}\left(S\right)$ is the solid stress tensor, ${\overset{↔}{T}}_{H}=-p\overset{↔}{I}+\eta \left(\nabla u_{f}+\nabla u_{f}^{T}\right)$ the hydrodynamic stress tensor, and 〈T↔E〉=12ε1Re(EE*−12E⋅E*I) the time-averaged Maxwell stress tensor evaluated along the particle surface on the liquid side.

A slip velocity appears on the surface of conducting membrane, while a continuity of the system speed is maintained at the liquid/PS interface:

(14) uf=us+ε12η(1+δ)Re((ϕ˜1−ϕ˜3)(E˜1−E˜1⋅n⋅n)*)uf=us

where $u_{s}\;=\;\partial S/\partial t$ is the local velocity of the Janus ME.

### 2.3. Numerical Simulation

A commercial software package, Comsol Multiphysics (version 5.3a, COMSOL Inc., Stockholm, Sweden), is used herein for numerically calculating the transient motion behavior of the asymmetric Janus cylinder immersed in aqueous electrolyte driven by an external AC forcing. On account of its time-dependent nature, strong fluid-structure interaction has to be incorporated into the simulation model. The 2D computational domain (Figure 1d) is virtually a x-z cross section of the 3D schematic diagram displayed in Figure 1a, which consists of an array of circular Janus ME freely suspended in a straight liquid channel sandwiched between a pair of AC-powered parallel electrode plates. The specific simulation procedure is as below.

(1) At first, we compute the Laplace equation (Equation (2)) for the three separate regions, including the bulk electrolyte, PS material, and the thin gold film, which is subjected to those conjugating conditions (Equations (3)–(5)) at different sharp material interfaces. In the meantime, AC voltage phasor is prescribed on the oppositely polarized metal plates (Equation (7)), and the normal potential gradient vanishes on both ends of the calculation domain (Equation (8)).

(2) Once the AC potential is known, it is then possible for us to get the knowledge of the particle–fluid interaction by computing Equation (9) in the solid region and Equation (12) in the liquid region, respectively. Stress balance incorporating the electrostatic force is imposed at the particle/liquid interface (Equation (13)). The speed of movement is continuous on the insulating surface of the Janus cylinder (Equation (14)), while a sharp ICEO slipping appears on the gold coating layer (Equation (14)). In this way, the combined effect of ICEO slipping and DEP force on the electrokinetic behavior of the Janus ME array is naturally included in our simulation model.

(3) Once the solid motion and flow field due to the action of AC electrokinetics are known, a particular moving mesh scheme is used to trace the transient evolution of the solid/liquid contact interface, with a request of the mesh velocity being equal to the computed particle velocity in the normal direction of the surface of the Janus ME. Once the structural interface moves more or less, all the field variables have to be recalculated with the simulation results regenerated incessantly by repeating (1)–(2) in the next round of numerical computation. Consequently, the calculation time step should be orders of magnitude shorter than the characteristic time scale of the solid and fluid mechanics, which is cautiously defined in the settings of the solver.

Since the AC electric field is complex in essence, while other field variables are real numbers, the AC electrostatic potential, and the field-induced fluid-solid interaction are solved with a transient solver in a segregating coupled way. The size of mesh on the particle surface is required to be no more than one-twentieth of the diameter of the rigid Janus cylinder, which remains a circular shape in the 2D domain all the time. This meshing scheme leads to 30,000–90,000 triangular grids in the 2D space, as determined specifically by the size and position of the Janus ME of interest. A stop condition is imposed to the transient solver, and dictates that the simulation process comes to a controlled halt once the leftmost (or the rightmost) end of the Janus ME approaches the entrance (or the exit) of the device channel of a rectangular shape. Please refer to Table 2 for the boundary conditions, and Table 3 for the simulation parameters pertinent to the fluidic device taking a Janus ME array as the functional microelectrodes.

## 3. Results and Discussion

### 3.1. Model Validation

Before making any quantitative calculations, we should demonstrate the validness of current physical model for dipolophoretic motion of asymmetric colloids at a given voltage. On the one hand, in the low-frequency limit, the Janus cylinder with an initial azimuth angle *θ*_0_ = 50° relative to the horizontal direction first rotates counterclockwise by ICEP torque, which makes its polar interface dividing the two hemispheres of different polarizability align well with the imposed field lines. Then, it moves almost unidirectionally upstream in the direction of its insulating PS end. Lateral motion occurs at the same time, however, since ICEO vortices on the particle surface near a conducting wall will always attract it to approach the ideally polarizable surface (See Supporting Video S1).

On the other hand, at frequencies beyond the inverse Debye relaxation time of the liquid bulk, the inclined Janus ME will still rotate counterclockwise by ER torque and achieve an identical alignment, while with its conductive end moving ahead toward downstream (see Supporting Video S2). Under this situation, however, no evident deviation of the particle from the channel centerline can be observed, since the phenomenon of particle-wall DEP interaction is a near-field effect in stark contrast to the far-field ICEO streaming flows in the low-frequency condition. In this sense, the effectiveness of the simulation model developed herein is proved on a qualitative level. On this basis, we then make a detailed investigation on the AC electrokinetic behavior of an individual Janus cylinder or an array of Janus ME, by conducting direct numerical simulation incorporating the surface-coupled fluid–structure interaction with a transient solver.

### 3.2. Directed Electrokinetic Transport of Janus Entity in a Wide Frequency Range

#### 3.2.1. AC Electrokinetics under the Low- and High-Frequency Limits

**Figure 2:**
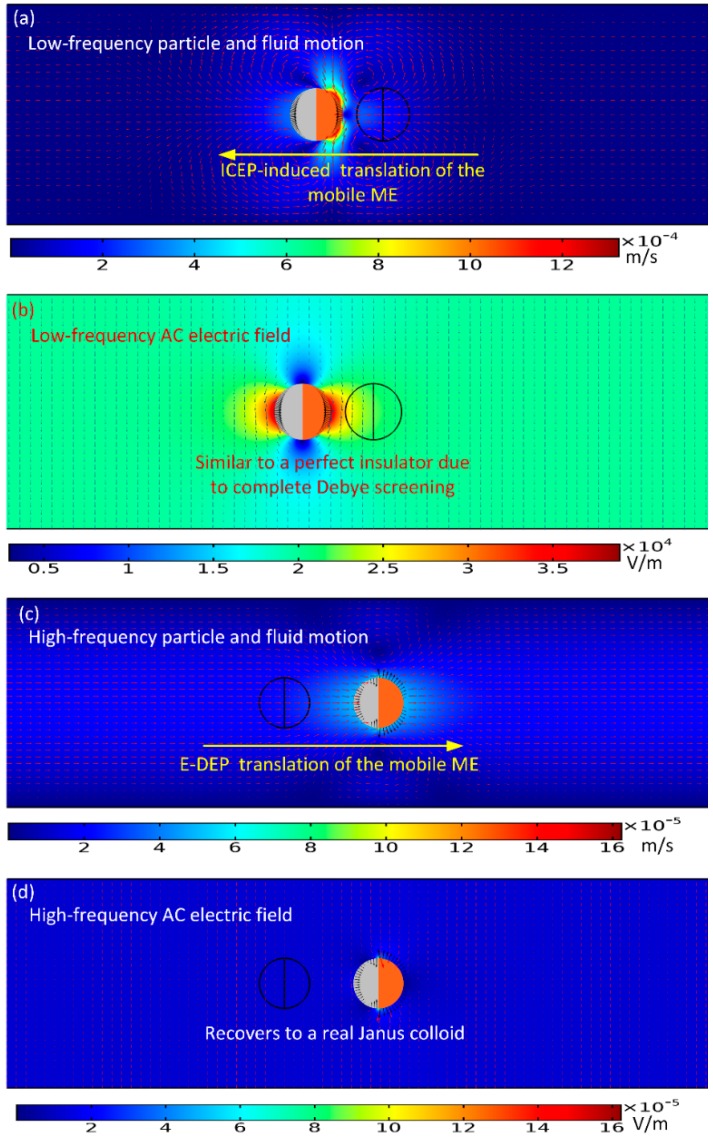
A simulation illustration of the electrokinetic delivery behavior of the Janus ME at distinct field frequencies under a given voltage amplitude of 1V. (**a**,**b**) In the low-frequency limit of *f* = 100 Hz and at a powering time of *t* = 0.1 s (See Supporting Video S1), (**a**) a surface and arrow plot of the ICEO-induced electrohydrodynamics (EHD) flow field and particle motion, and (**b**) an arrow and surface plot of the electric field around the Janus colloid of a symmetric induced dipole due to complete Debye screening on the conducting end. (**c**,**d**) In the high-frequency limit of *f* = 100 MHz and at a powering time of 1 s (See Supporting Video S2), (**c**) a surface and arrow plot of the ego-dielectrophoresis (e-DEP)-induced electrokinetic (EK) flow field and particle motion, and (**d**) an arrow and surface plot of the electric field around the Janus colloid of an asymmetric induced dipole due to the occurrence of electrochemical ion relaxation. The black arrows on the surface of the Janus particle (JP) in Figure 2 denote the interfacial Maxwell stress of a second-order voltage dependence.

From now on, we focus on the directed transport behavior of a single Janus mobile electrode (ME), with the simplest configuration wherein its polar interface is in the direction of the external AC field lines at the early stage, as shown in Figure 2a–d. In the computation, suitable geometry dimension is selected for the electrokinetic chip: *L_C_* = 300 μm, *H_C_* = 50 μm, *R* = 6 μm, *λ_D_* = 37.6 nm, along with the following physicochemical properties of the liquid and solid materials: *σ_f_* = 0.001 S/m, *σ_gold_* = 107 S/m, *σ_ps_* = 0 S/m, *C*_0_ = 0.018 F/m^2^, *ε_f_* = 80ε_0_, *ε_ps_* = 3ε_0_, *ε_gold_* = 10ε_0_, *τ_RC_* = *C_D_R*/*σ_f_*(1 + *δ*) = 5.5 × 10^−5^ s, *τ_MW_* = *ε_f_*/*σ_f_* = 7 × 10^−7^ s, and *η* = 0.001 Pa·s. The aqueous suspension is supposed to possess an electric conductivity of 0.001 S/m, giving rise to an inverse Debye relaxation time *f_MW_* = 1/2π*τ_MW_* = 225 kHz of the bulk electrolyte, as well as a reciprocal RC time constant *f_RC_* = 1/2π*τ_RC_* = 2.9 kHz for electrochemical polarization of the IDL at the membrane/suspension interface (Table 1 and Table 3).

To begin with, we numerically studied two limiting cases of this subject, namely, the low-frequency ICEP delivery (Figure 2a,b), and the high-frequency e-DEP transport (Figure 2c,d) affected by a uniform AC electric field. In the parameter settings, the AC voltage amplitude is fixed at 1V. The electric field strength equals 20 V/mm, which is on the same order of magnitude with that applied in typical ICEO experiments.

As for the low-frequency situation, the field frequency is 100 Hz, being much less than the inverse RC time scale *f_RC_* = 2.9 kHz for the field-induced Debye screening on the ideally polarizable surface of the conducting film. In this situation, the applied field lines bend across the particle/electrolyte interface around both hemispherical sides, in that the induced double layer (IDL) can be fully developed and then perfectly screen the normal field gradient emitted from the polarized gold membrane, as displayed in Figure 2b. As a consequence, the electric field pattern for the Janus cylinder at low frequencies is similar to that of a perfect insulating particle due to the occurrence of complete Debye screening on the conducting end. As shown in Figure 2a, since there is enough time for the bipolar counterions to amass within the thin boundary layer on the membrane surface, they are actively acted by the same frequency tangential AC forcing to engender a nonlinear ICEO vortex flow field around the conducting end, which even survives after time-averaging under AC (Figure 1b). ICEO slipping fluid motion injects from both the top and bottom ends, and then ejects selectively into the bulk suspension around the equatorial plane, resulting in the formation of a pair of ICEO micro-vortices in opposite rotating directions.

The induced-charge electro-osmotic flows, which originate from the action of an externally imposed AC electric field on its own induced charge within an induced double layer (IDL) on a polarizable solid surface immersed in electrolyte, won’t converge. Instead, the flow streams are coming outward to form vortices (Figure 1b). This type of vortex flows is well documented in pioneering literatures, wherein the earliest researches on DC/AC-induced ICEO vortex flow field on the sharp corner singularity of dielectric blocks of a small but finite permittivity comparable to that of water suspension were reported [78,79,80]. These are indeed great works on time-averaged ICEO streaming vortices on dielectric solid surfaces (rather than ideal metal conductors).

Recently, we have also investigated a time-averaged ICEO vortex flow adjacent to the sharp-corner-singularity of leaky dielectric blocks of both a finite conductivity and permittivity in external time-harmonic AC forcing [81]. In this paper, under the thin layer approximation and small double-layer voltage drop, we deal with the IDL as a thin capacitive skin induced at the dielectric wall/electrolyte interface by the applied AC field. The interaction of the imposed tangential AC forcing with its own induced bipolar Debye screening charge within the IDL gives rise to a pair of ICEO vortex in counter-rotating directions around the corner of the dielectric wall. Besides, if an additional DC component is applied across the channel length direction, linear DC electroosmotic (DCEO) pumping and DC-ICEO vortex flow appear at the same time. Once the DC voltage is much larger than the AC counterpart, an evident unidirectional DCEO flow would superimpose on the ICEO vortex flow pair induced by the combined AC and DC forcing, which has a tendency to flatten out the upstream ICEO vortex while with the swirling shape of the downstream ICEO vortex keeping almost unvaried. To this end, according to the Newton third law, the ICEO vortex flow profile will propel the Janus cylinder to move in the direction of the insulating end, as indicated in Figure 2a wherein the nearly symmetric dipole moment is delivered by ICEP forcing toward upstream for a powering time of 0.1 s.

For the sake of comparison, we then increase the imposed field frequency sharply from 100 Hz to 10 MHz. In this high-frequency limit, the IDL has already been short circuited by the constantly varying displacement current through it, so there is no longer asymmetric ICEO streaming flow around the solid entity. Since the permittivity effect dominates within the high frequency range, the insulating PS end still is less polarizable than the surrounding medium due to its lower dielectric permittivity compared to that of water. Nevertheless, the other hemisphere coated with thin gold membrane recovers to the intrinsic role of an ideal conductor on account of the incomplete capacitive charging of the IDL. In this way, the dipole moment of the conducting end is now positive, whereas the insulating end is still negative, which leads to the formation of an asymmetric induced dipole moment of the Janus ME (Figure 2d). Although the background electric field is uniform, the inhomogeneous dipole induced by the external field generates a strong asymmetry in electric field distribution around the particle. The action of the localized anisotropic field gradient due to the induced dipole acts on the asymmetric particle dipole moment itself, resulting in a new AC electrokinetic phenomenon called “ego-DEP (e-DEP)”.

The schematic diagram of the interfacial induced charge and the net nonlinear Maxwell stress is qualitatively shown in Figure 1c, which helps us to understand the physics of e-DEP more easily to a great extent. From Figure 1c, the electrical force in the direction of the polar interface vanishes considering a symmetry in vertical polarization, while the net electrokinetic stress in perpendicular orientation to the polar interface has a net effect in trying to push the Janus ME to move horizontally in the direction of the conducting end. Besides, since both the induced surface charge (free plus bond) and localized field gradient change direction within each half AC cycle, the net e-DEP force survives well after time-averaging in harmonic forcing, and can thereby constantly transport the asymmetric dipole moment towards the conducting end. This can be evidenced by the simulation result in Figure 2c wherein the Janus ME moves downstream due to a net electrical stress that points in the direction of the conducting end.

To this end, it has been demonstrated that the Janus entity acting as active ME can be either delivered by ICEP rotating vortex at low frequency or transported by e-DEP in high frequency limit.

#### 3.2.2. Electrokinetic Motion in a Wide Frequency Range

Although we have discovered a Janus ME can move electrokinetically in both low and high frequency limit. AC electrokinetic behavior in the intermediate frequency range is still unclear, however, so it is necessary for us to address this issue immediately. A parametric simulation study is then carried out to make it clear how the Janus cylinder makes a response to the applied AC forcing within a broad frequency range from 100 Hz to 100 MHz. Such an elaborate selection of the frequency limits is plausible, in that 100 Hz is much lower than the inverse RC time constant for capacitive charging of the IDL at the membrane/suspension interface, and 100 MHZ is much higher than the Debye relaxation frequency of the liquid bulk. This specific range defined by the lower and upper limits covers effectively the frequency band for any possible nonlinear electrokinetic phenomenon to occur.

**Figure 3:**
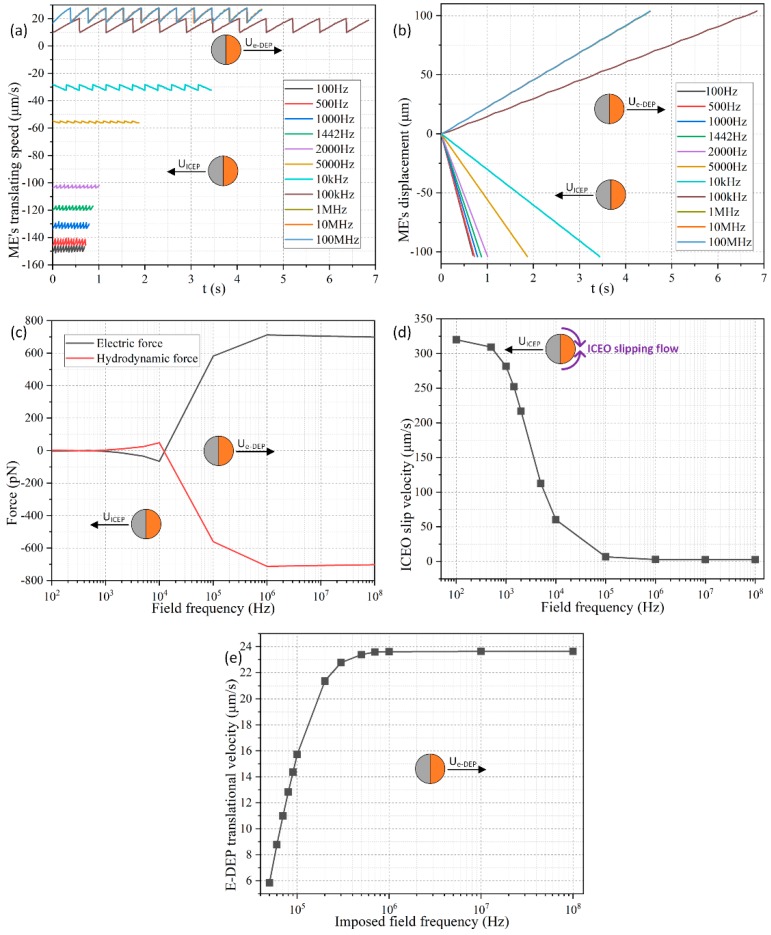
Influence of the imposed AC field frequency on the electrokinetic transportation behavior of an individual Janus ME with a diameter of 12 μm. (**a**) Time-dependent ME’s translating speed under different signal frequencies for a same total displacement of 105 μm. (**b**) The horizontal displacement of the Janus colloid as a function of time in a wide frequency range. (**c**) The balance between the electric force and hydrodynamic force acting on the Janus entity as a function of the field frequency. (**d**) Frequency-dependent ICEO slipping velocity on the surface of the conducting end. (**e**) e-DEP-induced JP translating speed as a function of the imposed field frequency from 50 kHz to 100 MHz. It is noteworthy that ICEP dominates in the frequency range below 50 kHz, so the start threshold frequency is set at 50 kHz in Figure 3e.

As shown in Figure 3a, the direction of movement of the Janus ME is negative, and points to the insulating end from 100 Hz to 10 kHz, because ICEP propulsion dominates its motion behavior at frequencies around the inverse RC time scale *f_RC_* = 2.9 kHz for electrochemical polarization of the membrane in direct contact with the aqueous electrolyte. The negative translational velocity equals −150 μm/s at 10 Hz, and gradually decreases to −30 μm/s with an increase of frequency to 10 kHz, which is due to an electrochemical ion relaxation of the double layer charge that weakens ICEO slipping velocity under higher excitation frequencies (Figure 3d).

On the other hand, for the imposed field frequency no less than 50 kHz, the moving direction of the Janus cylinder alters from negative to positive (Figure 3e). Under this circumstance, there is not enough time for the charged counterions to accumulate effectively within the thin boundary layer, so that the phenomenon of ICEO fades away, and the particle dipole moment recovers to an asymmetric one when considering there is no longer Debye screening on the conducing hemisphere. In this way, the e-DEP-enabled electrokinetic force governs the motion of the Janus cylinder, which increases with frequency and attains a plateau of 24 μm/s at frequencies beyond 1 MHz (Figure 3e). Any further increase of the field frequency exceeding 1MHz would no longer alter the magnitude of the induced dipole moment, in that the gold membrane has a conductivity of 10^7^ S/m about ten orders of magnitude larger than that 10^−3^ S/m of the liquid suspension. Though the e-DEP-induced particle translating movement +24 μm/s (Figure 3e) within the high frequency range is smaller than −150 μm/s caused by ICEP in low frequency limit, it is still observable and provides a supplementary transport direction. The discovery of the effect of e-DEP enriches the AC electrokinetic behavior of Janus entity in a uniform electric field, and allows a direction-controllable delivery within a wide frequency range.

Meanwhile, the transient moving speed behaves as an oscillating profile as time advances, which arises mainly from a dynamic balance among the various forces exerted on its surface, including active ICEP propelling, interfacial nonlinear Maxwell stress, Stokes drag force, as well as the stress from the rigid PS body, as can be clearly proved in Figure 3c wherein the electric force and hydrodynamic force always counterbalance one another. Even so, the horizontal displacement exhibits a monotonous growing trend as a function of time whatever the imposed field frequency is (Figure 3b). As a consequence, we pay less attention to the transient fluctuation in the speed of movement, and mainly focus on the time-averaged translational speed from now on.

### 3.3. Parametric Study for the Low-Frequency Induced-Charge Electrophoretic (ICEP) Translation

In this section, the field frequency is fixed at *f* = 100 Hz, which is well below the RC charging frequency of the IDL at the ideally polarizable surface of the gold membrane. In such a low-frequency limit, we discerned the effects that the dimension of the Janus entity and the magnitude of AC voltage have on the resulted particle translating kinematics.

**Figure 4:**
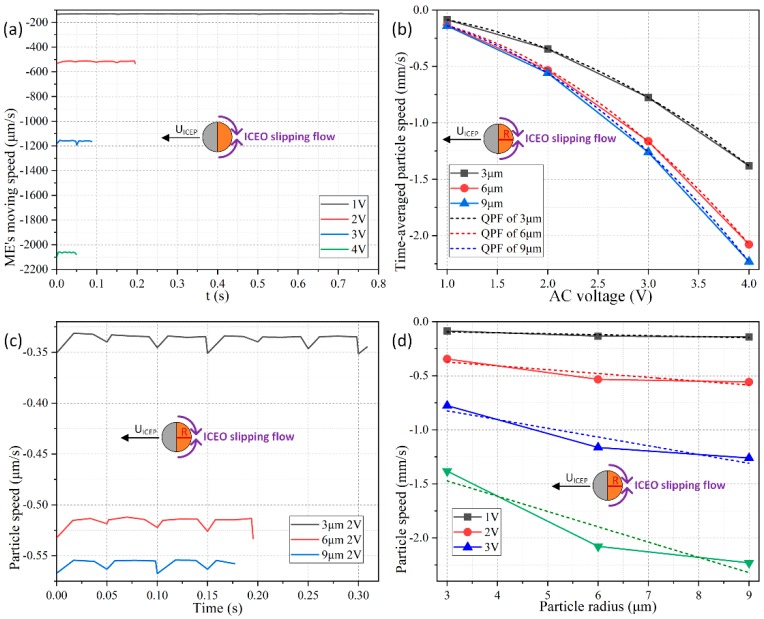
Parameter-dependence study of the low-frequency ICEP translation of the Janus ME in the direction of the insulating end at 100 Hz. (**a**) Moving speed of the Janus colloid as time elapses under different AC voltages at a given particle radius of 6 μm. (**b**) Voltage-dependence of the time-averaged particle’s translating speed for different colloid radius as well as their quadratic polynomial fitting (QPF) curves. (**c**) Moving speed of the Janus colloid as time elapses for different particle radius at a given voltage of 2 V. (**d**) Diameter-dependence of the time-averaged particle’s translating speed for different voltage amplitude as well as their linear polynomial fitting (LPF) curves.

As for the influence of the applied voltage, the colloidal radius is 6 μm, and the signal amplitude is arbitrarily swept from 1 V to 4 V with a fixed discrete interval of 1 V. As shown in Figure 4a, the ICEP motion in the direction of the insulating PS end has a translating speed that grows quadratically with the applied voltage, namely, *U_ICEP_* ∝ *V*^2^ and thereby much less time is required for the Janus cylinder to cover an identical horizontal distance of 100 μm when the voltage is enhanced from 1 V to 4 V as other conditions remain unchanged. On the other hand, under a given voltage amplitude of 2 V, although the negative speed of movement also increases with increasing particle diameter, this growing trend has a power law with the exponent less than one as a function of colloidal dimension, that is, *U_ICEP_* ∝ *R^α^*, with 0 < *α* < 1. In this way, according to our direct simulation study, *U_ICEP_* ∝ V^2^R^α^ (0 < α < 1) is against the scaling rule of ICEO around a metal particle, *U_ICEP_* ∝ *V*^2^*R*, given by Bazant and Squires for an infinitely large liquid domain [82]. The reason behind this particular aberration is originated by a vertical confinement effect due to the finite space of current fluidic chamber, which merely has a height of 50 μm. Under this circumstance, as long as the particle diameter is no less than one-tenth of the channel vertical dimension (50 μm/10 = 5 μm), the vertical confinement effect will attempt to suppress the healthy development of the ICEO slipping vortex flow field adjacent to the conducing side (Figure 2a), which counteracts the enhanced induced polarization of the gold membrane from a larger particle radius, so that the scaling characteristic of the ICEP transporting speed eventually manifests as *U_ICEP_* ∝ *V*^2^*R^α^* with 0 < *α* < 1.

The coupled interaction between the colloid dimension and AC voltage for the electrokinetic behavior of the Janus entity is displayed in Figure 4b,d as well, wherein we provide polynomial fitting for each data curve. The voltage-dependent moving velocity agrees well with the quadratic polynomial fitting (QPF) in Figure 4b, demonstrating the effectiveness of *U_ICEP_* ∝ *V*^2^. Nevertheless, the radius-dependent translating speed is always lower than the linear polynomial fitting (LPF) in Figure 4d, which witnesses the correctness of the scaling law of *U_ICEP_* ∝ *R^α^* (0 < *α* < 1).

To this end, within a fluidic chamber, in which as long as there is one dimension that is comparable to the size of the suspended particles, it is no longer convenient to increase the ICEP motion by enlarging the particle diameter due to the action of a confinement effect. Alternatively, enhancing the applied voltage serves as a more ideal way for accelerating the unidirectional delivery speed of the micro Janus cylinder in electrolytes.

### 3.4. Parametric Study For High-Frequency e-DEP Translation

To confirm the existence of e-DEP and its dependence on some key parameters, the field frequency is subsequently enhanced to 10 MHz. Under such high frequency excitations, both the electrochemical polarization at the membrane surface and the associated ICEP propulsion effect decay to zero due to electrochemical ion relaxation. So, only Maxwell-Wagner interfacial polarization plays an important role in high-frequency electrokinetic behavior of the Janus cylinder, with the schematic vividly shown in Figure 1c. The gold film recovers to its original role of a perfect conductor in the absence of conspicuous Debye screening. Consequently, symmetry breaking in the induced dipole moment of the Janus colloid takes place. Although a uniform background AC electric field is imposed along the channel depth direction (Figure 1a), the field strength is greatly perturbed to become quite uneven around the particle (Figure 2d), which acts on the asymmetric dipole induced by the field itself, so as to enable a continuous translating motion in the direction of the conducting end (Figure 2d).

**Figure 5:**
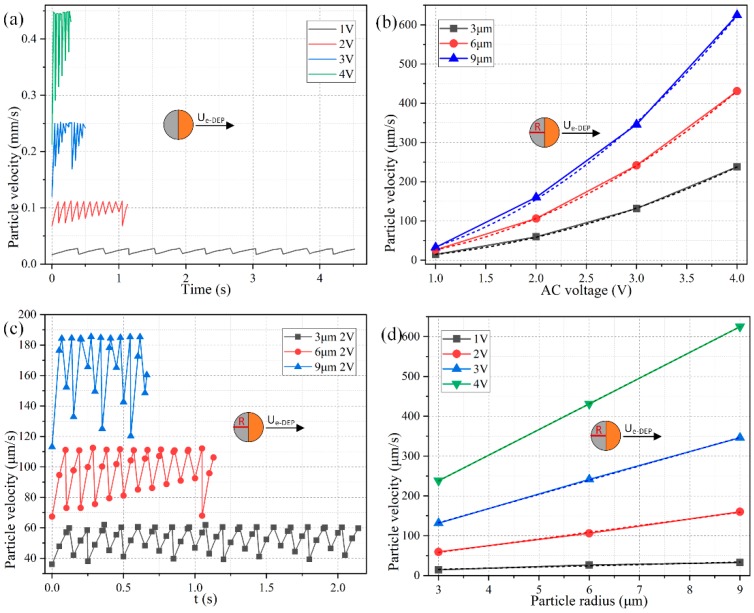
Parameter-dependence study of the high-frequency e-DEP translation of the Janus ME in the direction of the conducting end at 10 MHz. (**a**) e-DEP moving speed of the Janus colloid as time advances for different AC voltage with a given particle radius of 6 μm. (**b**) Voltage-dependence of the time-averaged particle’s translating velocity for different colloid radius as well as their quadratic polynomial fitting (QPF) curves. (**c**) e-DEP moving speed of the Janus colloid as time advances for different particle radius at a given voltage of 2 V. (**d**) Diameter-dependence of the time-averaged particle’s translating speed for different voltage amplitude as well as their linear polynomial fitting (LPF) curves.

As shown in Figure 5a, under a given colloid radius of *R* = 6 μm, as the voltage amplitude increases from 1 V to 4 V, the time-averaged moving speed of the Janus mobile electrode (ME) grows from 26.5 μm/s to 430 μm/s, implying a quadratic growth trend of the e-DEP-induced speed of movement as a function of the AC forcing. Namely, the scaling law of DEP motion in an inhomogeneous electric field above a microelectrode array, *U_e-DEP_* ∝ *V*^2^, is still applied for e-DEP motion of the Janus ME affected by a uniform potential gradient, when considering its nonlinear nature that the localized field gradient interacts with its own induced asymmetric particle dipole moment.

On the other hand, the dependence of the e-DEP velocity on the dimension of the particle is somewhat counterintuitive. A shown in Figure 5c, the particle velocity at 10 MHz from e-DEP is almost linearly proportional to the radius of the ME, namely, *U_e-DEP_* ∝ *R*, which differs from the typical DEP velocity scale of *U_DEP_* ∝ *R*^2^ in a field gradient. This disparity may be ascribed to the fact that, the convectional DEP motion is caused by a background field gradient, while the translating behavior of e-DEP is due to the action of a secondary field gradient around the particle actuated by a uniform AC forcing. As a consequence, the e-DEP speed obeys the scaling trait of *U_e-DEP_* ∝ *V*^2^*R*.

These conclusions can be also demonstrated in Figure 5b,d. As displayed in Figure 5b, the simulated data of the voltage-dependent e-DEP velocity (the solid lines) almost overlap with their QPF curves (the dashed lines). Unlike the vertical confinement effect that the finite channel height has on the ICEP translation in low-frequency limit (Figure 4d), this negative influence is not adaptable to the phenomenon of e-DEP at all. Even if the diameter of the ME increases to 18 μm for a channel height of 50 μm, the linear radius-dependence characteristic of the moving speed is still valid (Figure 5d). This clearly indicates the physical origin of e-DEP is electrokinetics (EK) but not electrohydrodynamics (EHD), since EK is usually not sensitive to a finite calculation domain, while EHD is more sensitive to the volume of the computational geometry.

It is worth noting that the speed of movement due to e-DEP at high frequencies (Figure 5b,d) is about one-third of that from ICEP within the low frequency range (Figure 4b,d). Even so, e-DEP may serve as a better method of choice for unidirectional transport of the Janus ME, in that the application of a high field frequency greatly eliminates the unwanted effects of electrochemical polarization and electrode erosion, both of which can cause potential damage to any biological content within the buffer.

### 3.5. Collection of Nanoscale Analyte Using an Individual Janus Mobile Electrode

On the basis of the fundamental studies in preceding sections, it is then interesting to test whether the direction-controllable AC electrokinetic transport of the Janus ME can be applied for sequential loading and collection of nanoscale biomolecules from the surrounding liquid medium, when it moves continuously in perpendicular orientation to the externally-imposed AC forcing.

**Figure 6:**
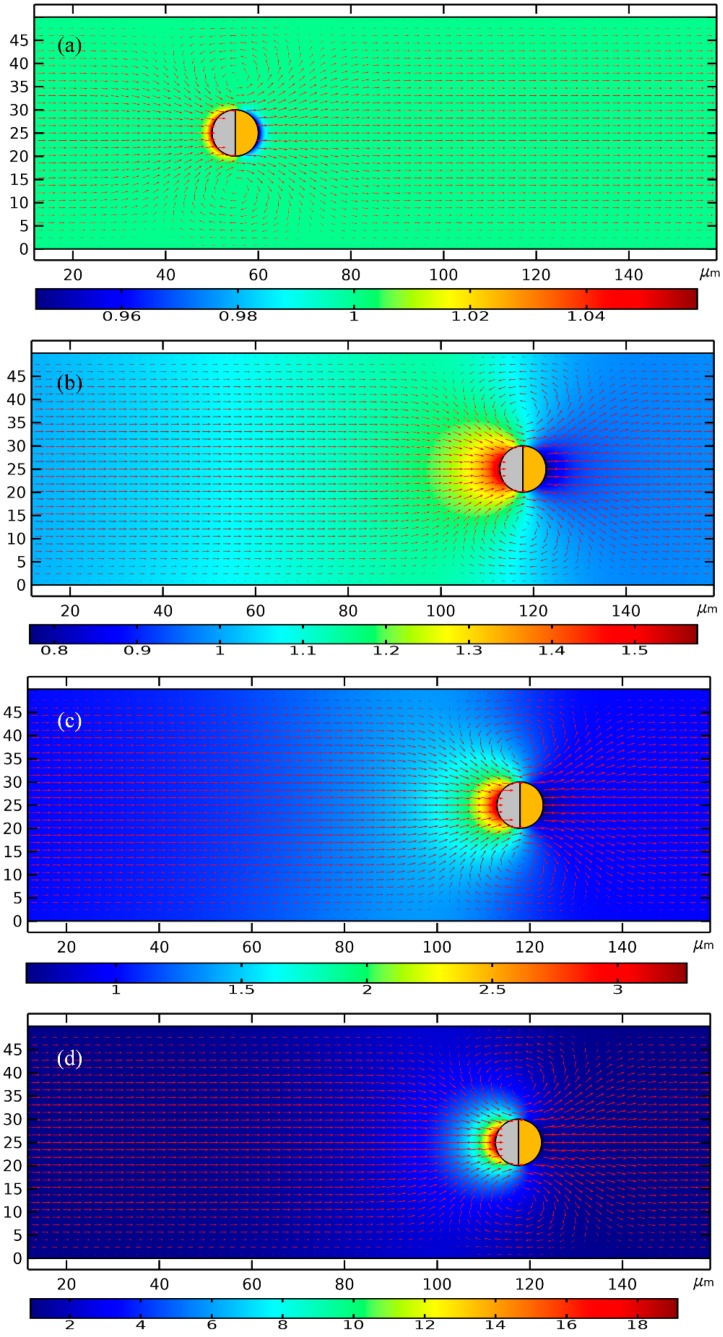
A simulation investigation of the application of the e-DEP-enabled unidirectional delivery of the Janus ME (PP) in simultaneous hydrodynamic loading and collection of the nanoscale biomolecules (SP) from the surrounding liquid suspension in the high-frequency limit. (**a**–**d**) An arrow plot of the hydrodynamic flow field due to the e-DEP motion of the Janus ME, and a surface plot of the concentration field of the biomolecules (unit: nM) inside the fluidic chamber at *f* = 10 MHz, (**a**) at *t* = 0 s, (**b**) at *t* = 2.7 s for *V_AC_* = 1 V, (**c**) at *t* = 1.2 s for *V_AC_* = 1.5 V, and (**d**) at *t* = 0.67 s for *V_AC_* = 2 V (See Supporting Video S4). In (**b**–**d**), the Janus PP has moved a same horizontal distance of 60 μm as compared to (**a**). Besides, with increasing voltage from (**b**) to (**d**), the concentrating factor of the SP increases, while the collection area reduces.

Trace molecule assumption is invoked here, namely, the concentration of the biological analyte is orders of magnitude lower than that of the ionic charge carriers (cations and anions) in the electrolyte solution. So, the total charge magnitude of the biomolecules is negligibly small, and its contribution to the charge conservation equation can be safely disregarded. In this sense, all the electric field equations in Section 2 are still valid under current situation. Since an external AC field is employed, the effect of linear electrophoresis time-averages to zero and can then be discarded. The dielectrophoretic (DEP) force of a nanoscale object is also trivial, as evidenced by the quantitative computation shown in Figure 6a. The analyte velocity induced by DEP force field is far below 1 μm/s, albeit the field gradient attains the order of O (10^4^–10^5^) V/m.

According to the analysis above, the concentration field of the biomolecules is calculated via a traditional convection-diffusion equation for dilute analyte:

(15) $$\frac{\partial c}{\partial t}+\nabla \cdot J=0\;\;\;J=u_{f}c-D\nabla c$$

where *c* is the concentration field of the charged biomolecules, *J* the volumetric flux density of the analyte, and *D* the analyte diffusivity of the secondary particle (SP).

The nanoscale biomolecules around the primary particle (PP) of the Janus ME is supposed to have a diameter of *d* = 2*r* = 100 nm, which is about two to three orders of magnitude smaller than the Janus ME, so they can be treated as secondary particles (SP). The thermal diffusivity of the biomolecule is 4.3 × 10^−12^ m^2^/s, according to the Einstein relation *D* = *k_B_T*/6*η*π*r*. Here, *k_B_* is the Boltzmann constant, and *T* = 293.15 K the ambient temperature. Impenetrable condition is imposed on the opposing electrode plates with zero normal mass flux. The left and right ends of the chamber are set as open boundaries. Early on, the biological SP is supposed to distribute uniformly within the chamber with a background concentration value of 1 nM.

**Figure 7:**
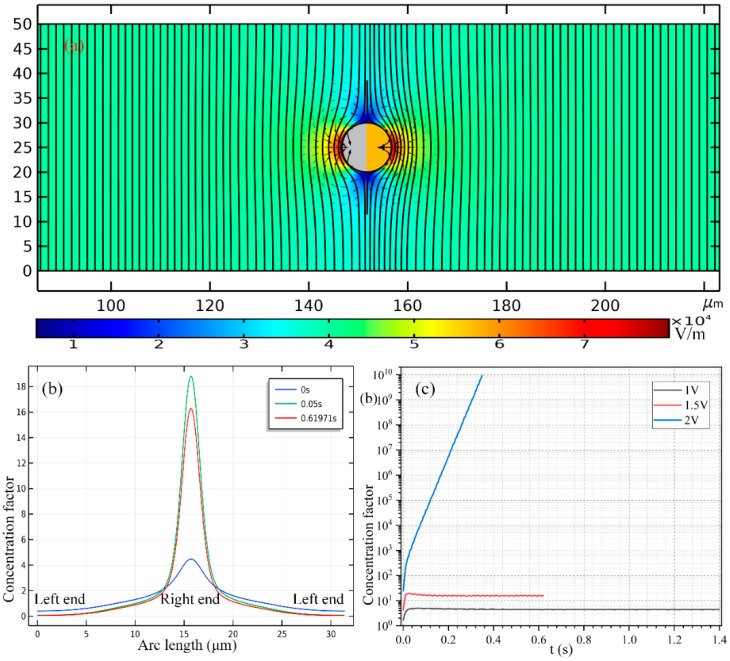
(**a**) The positive DEP velocity field of the nanoscale biomolecules dispersed in the liquid suspension around the Janus ME having an almost symmetric induced dipole moment in the low-frequency limit, which is on the order of 10^−10^–10^−7^ m/s and can thereby be ignored in comparison with the electroconvective flow effect. (**b**) The concentrating factor along the surface of the Janus PP at distinct time instants once the sinewave generator with an AC voltage of 1.5 V at *f* = 100 Hz is switched on. (**c**) The peak concentration factor of the target biomolecules on the surface of the Janus ME as a function of time for varying AC voltages at *f* = 100 Hz.

**Figure 8:**
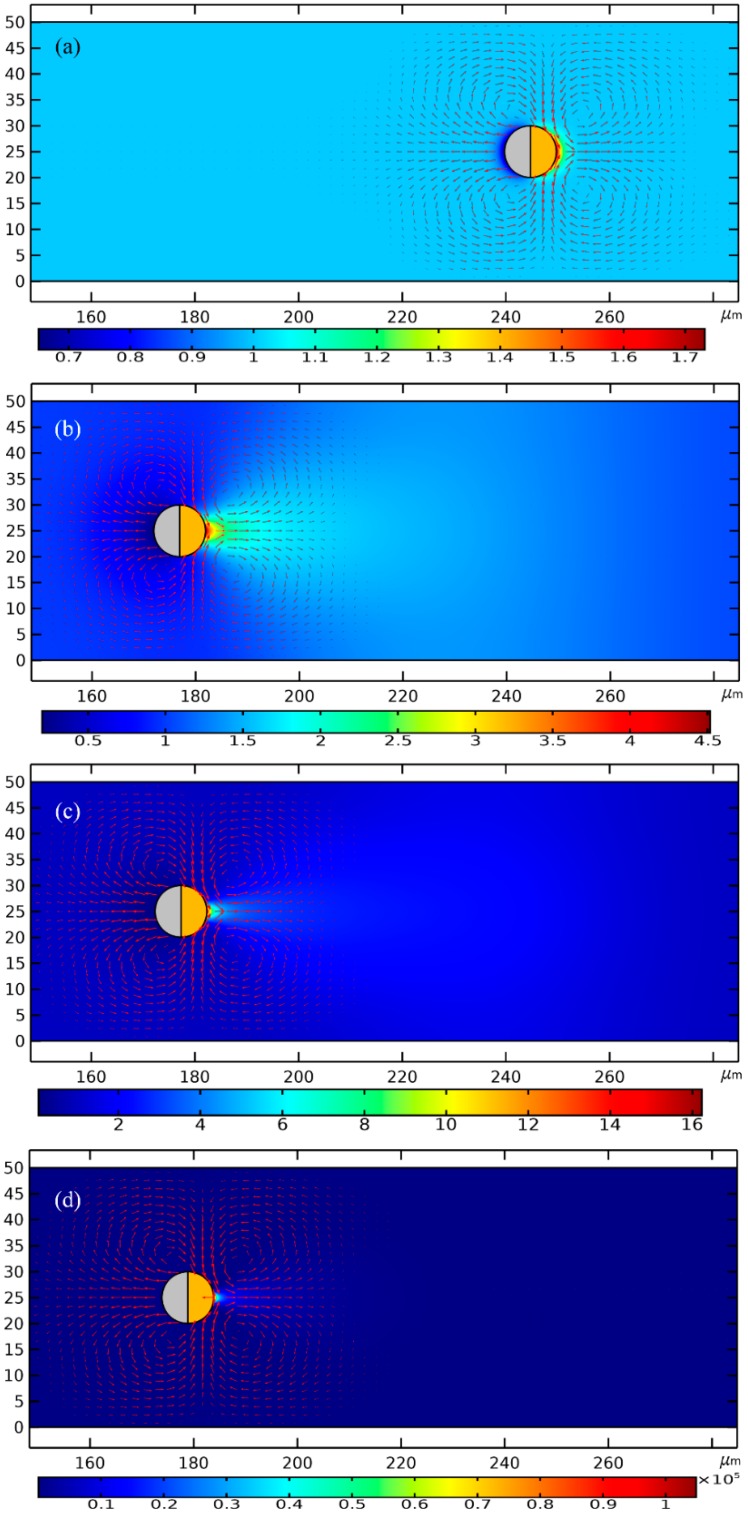
A simulation study of the application of the ICEP-enabled unidirectional delivery of the Janus ME (PP) in simultaneous electroconvective loading and collection of the nanoscale biomolecules (SP) from the surrounding liquid suspension in the low-frequency limit. (**a**–**d**) An arrow plot of the EHD vortex flow field around the ME, and a surface plot of the concentration field of the biomolecules (unit: nM) inside the fluidic chamber at *f* = 100 Hz, (**a**) at *t* = 0 s, (**b**) at *t* = 0.5 s for *V_AC_* = 1 V, (**c**) at *t* = 0.22 s for *V_AC_* = 1.5 V, and (**d**) at *t* = 0.17 s for *V_AC_* = 2 V (See Supporting Video S3). In (**b**–**d**), the Janus PP has moved a same horizontal distance of 70 μm as compared to (**a**). Besides, with increasing voltage from (**b**) to (**d**), the concentrating factor of the SP enhances sharply, while the collection area shrinks gradually.

**Figure 9:**
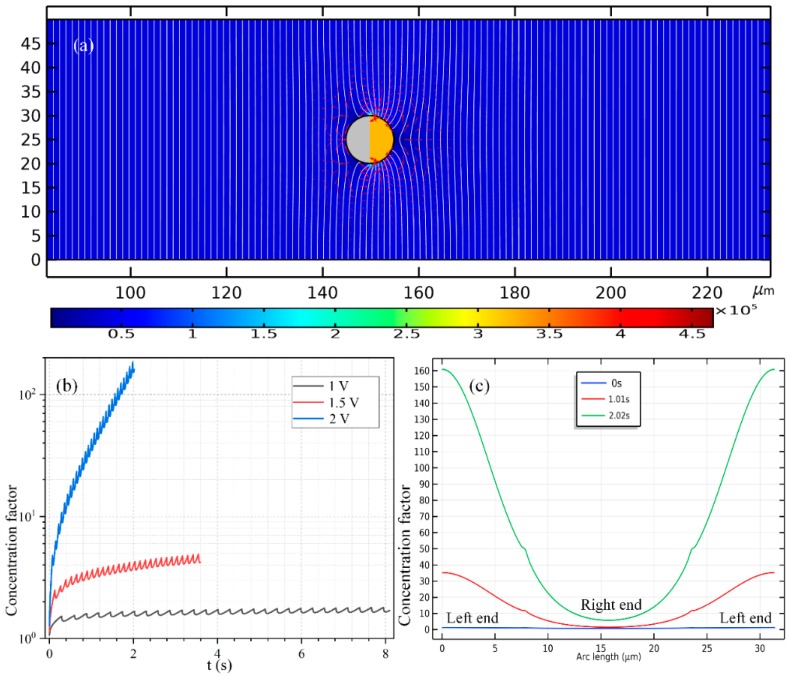
(**a**) The positive DEP velocity field of the nanoscale biomolecules dispersed in the liquid suspension around the Janus ME having an asymmetric induced dipole moment in the high-frequency limit, which is on the order of 10^−10^–10^−7^ m/s and can thereby be ignored in comparison with the hydrodynamic flow effect. (**b**) The peak concentration factor of the target biomolecules on the surface of the Janus ME as a function of time for varying AC voltages at *f* = 10 MHz, and the translating speed of the PP equals 23.46 μm/s for 1 V, 52.9 μm/s for 1.5 V, and 94 μm/s for 1.5 V. (**c**) The concentrating factor along the surface of the Janus PP at distinct time instants once the sinewave generator with an AC voltage of 1.5 V at *f* = 10 MHz is switched on.

With the above settings in the simulation software, the transient behavior of loading and collection of the biological SP is then investigated in terms of two distinct situations, including the low-frequency ICEP (Figure 7 and Figure 8) and high-frequency e-DEP delivery of the asymmetric PP (Figure 6 and Figure 9), respectively.

#### 3.5.1. Loading and Concentrating of SP by a Janus PP in Low Frequency Range

As shown in Figure 8, to enable a vivid visual clarification of the process of cargo loading, we present a surface plot of the SP’s concentration field and an arrow plot of the flow field within the liquid domain, along with the ICEP-driven delivery of the mobile PP in the direction of the insulating end towards the entrance. The Janus PP is released at x = 245 μm in the vicinity of the channel exit with a zero initial velocity (Figure 8a). On application of a low-frequency AC signal with a voltage amplitude from 1 V (Figure 8b), 1.5 V (Figure 8c) to 2 V (Figure 8d), ICEO vortex flow field is induced by the applied field preferentially on the surface of the conducing end (Figure 8a). The ME moves in the direction of the insulating end by ICEO propulsion on the membrane surface. As it transports via ICEP towards the entrance, the strong electrohydrodynamic slipping flow field of two oppositely rotating micro-vortices entrain the surrounding biomolecules, and make these SP collect on the rightmost end of the conducting membrane. In fact, this physical process is dynamic in nature, wherein the directed motion of PP and the collection of SP on some portion of the PP surface occur simultaneously, so that the same transient solver is always employed for the calculation of electric-field-mediated fluid-structure interaction and analyte mass transfer in the medium domain.

For the purpose of comparison, we plot the concentration field of SP and the position of PP at a same location x = 175 μm of the PP. Not only much less time is required for the Janus ME to cover an identical distance of 70 μm (245 − 175 = 70 μm), but the peak concentration of the SP collected by ICEO slipping on the conducting surface increases sharply as well with an enhancement of the applied voltage (Figure 8b–d).

In Figure 7b, we present the concentration profile of the biomolecules on the surface of Janus cylinder at three distinct time instants on switching the function generator on for a voltage of 2 V, including *t* = 0 s, 0.05 s and 0.61971 s. As we can see, with time elapses, the peak concentration increases at first, then begins to decrease at a critical time node, this varying trend is also clearly shown in Figure 7c. Even so, the width of the collection band shrinks monotonously with time (Figure 7b). In addition, though a smaller voltage induces a lower localized concentration factor of the biomolecules due to a reduced ICEO slipping flow field (Figure 7c), a voltage of 1 V gives rise to a larger collection area (Figure 7b) as compared to the case of both 1 V (Figure 8c) and 2 V (Figure 8d). This suggests us to make use of a moderate voltage amplitude for loading and collection of SP in real applications, so we can make a delicate trade-off between the practically realizable trapping area and the concentration factor.

#### 3.5.2. Loading and Concentrating of SP by a Janus PP in High-Frequency Limit

Since the high-frequency e-DEP motion reverses direction with respect to the low-frequency ICEP translation, the Janus PP is placed at x = 55 μm adjacent to the channel entrance before turning on the sinewave generator for high-frequency actuations.

Similar simulation results are presented in Figure 6 for the case of e-DEP as Figure 6 for the situation of ICEP, albeit the initial position of the mobile PP is adjacent to the entrance (Figure 6a) rather than the exit (Figure 6a). Under different voltage amplitudes but a same displacement of 60 μm, the concentration field of the biological SP and the electrokinetic flow field are compared in Figure 6b for 1 V, Figure 6c for 1.5 V and Figure 6d for 2 V. It can be seen that, an increasing voltage implies a higher electrokinetic flow rate caused by a larger particle e-DEP velocity, as well as a higher peak concentration ratio on the surface of the insulating hemisphere (Figure 6). The area of collection of the secondary nanoparticles diminishes as the voltage enhances (Figure 6), nevertheless, which is quite similar to the case of high-frequency excitation (Figure 8).

The time-dependent concentrating factor is provided in Figure 9b. The high-frequency growth trend, however, now manifests as a monotonous increasing trend once ignoring the localized secondary fluctuation (Figure 9b), which forms a stark contrast with the non-monotonous time-dependent variation in the low-frequency case (Figure 7d). In addition, the quantitative data of the concentration value of the biomolecules along the ME’s surface in Figure 9c also indicates that, the concentrating efficiency increases, while the area of the concentration belt decreases as the powering time advances.

The most notable difference between the two limiting cases of low- and high-frequency biomolecule loading is that: the swirling motion of the SP around the mobile PP is caused by ICEO whirlpools, which is EHD in essence (Figure 8); on the contrary, the main force that drives the nanoparticles to the surface of the insulating end is the fluid flow from e-DEP motion of the ME, which is of an electrokinetic origin, and this passive electrokinetic flow driven by high-frequency e-DEP (Figure 6) is much slower than the active electrohydrodynamic flow due to low-frequency ICEO slipping (Figure 8). As a result, the concentrating factor is much higher at low frequencies (Figure 7c), in which the active ICEO slipping flow around the constantly translating Janus ME brings large amounts of biomolecules to the conducting end from surrounding electrolyte (Figure 8d).

### 3.6. Continuous Collection of Biomolecules in Buffer Medium using an Array of Janus Mobile Microelectrodes

**Figure 10:**
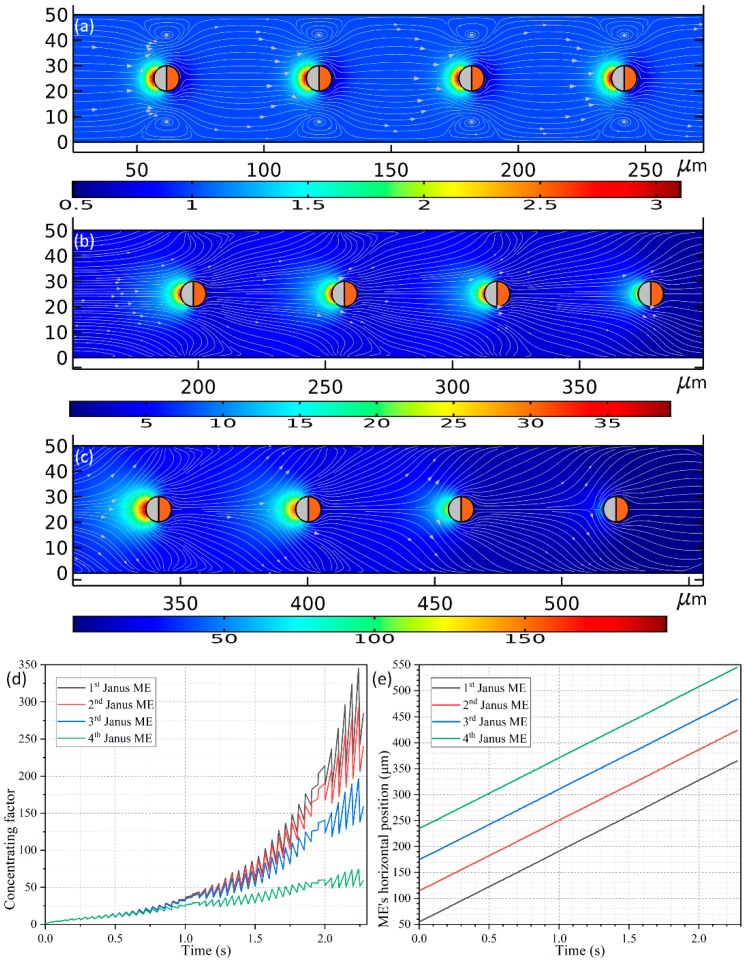
A simulation study of the feasibility of an array of Janus ME with four motile PP arranged along the channel centerline driven by e-DEP force in continuous collection of secondary biomolecules from a 0.1 S/m buffer medium with an AC voltage signal of 1.5 V and 500 MHz. (**a**–**c**) An arrowed streamline plot of the hydrodynamic flow field around the array of the Janus ME experiencing e-DEP force, and a surface plot of the concentration field of the biomolecules (unit: nM) inside the fluidic chamber, at (**a**) *t* = 0.05 s, (**b**) *t* = 1.05 s, (**c**) *t* = 2.1 s (See Supporting Video S5). (**d**) Temporal evolution of the concentrating factor of the target biomolecules on the surface of the sequential PP as the ME array moves via e-DEP motion. (**e**) Time-dependent horizontal location of the four motile electrodes as they transport unidirectionally towards downstream, from which a time-averaged e-DEP translational velocity of 136.318 μm/s is obtainable.

We then arbitrarily raise the liquid conductivity from the previous 0.001 S/m to 0.1 S/m to approach a more realistic biological culture condition. With such high ionic strength, ICEP decays almost to zero due to ion overcrowding inside the IDL. In this way, the Janus ME can only be delivered by the mechanism of e-DEP, which itself is not essentially limited by the steric effect and still work well at suitable excitation field frequencies. The enhancement of the solution conductivity leads to an augment of the inverse Debye relaxation time of the liquid bulk of *f_MW_* = 22.5 MHz. According to previous analysis, the imposed field frequency has to be lifted up to exceed this particular value in order for e-DEP to occur. In this sense, a sinusoidal voltage signal at *f* = 500 MHz and *V*_0_ = 1.5 V is imposed to the oppositely-polarized driving electrode pair for causing observable e-DEP-induced unidirectional motion of an array of Janus ME with 4 PP placed sequentially along the channel centerline (See Supporting Video S5). To accommodate the increase in the size of ME array, the channel length is correspondingly enlarged from 300 μm to 600 μm (Figure 10a–c).

As shown in Figure 10a, four spherical PP with the line connecting their centers being parallel to the channel length direction are released around the inlet. All the PPs have an identical diameter of 10 μm as used in Section 3.5, and the distance between the nearest ends of adjacent ME is *L_G_* = 50 μm. On application of an external harmonic AC forcing of *f* = 500 MHz and 1.5 V to the conducting plates, the four Janus ME move unidirectionally towards the channel outlet at an almost equal translating speed of 136.318 μm/s due to the action of e-DEP mechanism, which is much quicker than that of 52.9 μm/s utilizing an individual ME. The reason behind this acceleration can be vividly accounted for by a making a direct comparison between Figure 6 and Figure 10a–c. As shown in Figure 10a–c, the hydrodynamic convection induced by each Janus ME is in the same direction, and thereby can be effectively superimposed to some extent, which has closing bearing on the interparticle separation (not shown). An enhanced electroconvection helps to transport the ME array much more effectively under the influence of e-DEP, and simultaneously gives rise to a much higher concentrating factor, which is even beyond 100-fold (Figure 10d) in comparison with four-fold induced by a single motile electrode (the red line in Figure 9b).

A fact of interest is that, the peaking concentrating performance evolves differently for the four Janus ME (Figure 10d). Early on, right after switching the functional generator on, the value of concentrating index of the biomolecules grows consistently for all the motile electrodes before *t* = 0.75 s. With the advance of time, the SP concentration factor attains 25-fold on the surfaces of the ME array. Nonetheless, since the PP in the upstream section has a higher chance to capture the dispersed SP, the growth trend of the concentrating efficiency diverges from now on, and more nanoparticles would be trapped on the mobile electrodes they pass through first. For such, although an increasing trend is found for all the Janus ME, the peak concentrating factor decreases step by step as the biomolecules travel from the 1st ME to the 4th ME (Figure 10d). And this phenomenon is most evident in the latest stage as displayed in Figure 10c, implying it serves as a potential method for constructing concentration gradient generators for various on-chip biomedical applications.

In this particular case, the gap size *L_G_* = 50 μm between the mobile electrodes keeps constant while they move by e-DEP. In preliminary testing, however, we also simulated the cases in which the interelectrode separation *L_G_* = 10–40 μm, and we found that there is active electrokinetic and electroconvective interactions among them, which behaves as a rather complex nature. So, we do not focus on this subject in the current analysis, but can still roughly conclude that, if the interelectrode gap is no less than 10 times the ME’s radius, i.e., L_G_ ≥ 10 × R, the kinematic interaction between neighboring Janus entities will be negligibly small and thereby the gap between the electrodes will be maintained while moving them.

### 3.7. Scaling Analysis of the Stable ICEP Motion In Low-Frequency Limit

An approximate analytical solution of the scale of the induced zeta potential is given by:

(16) $$\zeta \approx \frac{E_{B}\cdot R}{\left(1+\delta \right)\left(1+j\omega \tau_{\mathrm{RC}}\right)}$$

where *E_B_* denotes the applied electric field strength, and *R* the radius of ME, *δ* the surface capacitance ratio, *ω* the angular field frequency, *τ_RC_* = *C_D_R*/*σ_f_*(1 + *δ*) the RC time constant for capacitive charging of the induced double layer.

Conventionally, ICEP insists well in a so-called low-voltage limit, in which the voltage drop across the diffuse part of the induced double layer (IDL) is no more than about a threshold value of $\zeta_{0}$ = 1.5 V. Note that δ is usually far less than 1, and thereby we have the scaling expression of AC voltage amplitude in DC limit:

(17) $$V_{\mathrm{AC}}\leq \frac{\zeta_{0}H_{C}}{R}$$

For the geometry size of *H_C_* = 50 μm and *R* = 6 μm used in current analysis, the AC voltage magnitude should be no more than 12.5 V, that is, V_AC_ ≤ 12.5 V according to the preceding equation, which is in good agreement with the range of V_AC_ (V_AC_ ≤ 4 V) applied in the parametric study of current work. Under such circumstances, the non-uniform surface electrokinetic transport of counterions within the Debye screening layer becomes quite limited, so do the resulted ion concentration polarization (ICP) phenomenon and electroosmosis of second kind that behaves as a strong electrokinetic eddy in the depleted region, due to a sufficiently small Dukhin number (a physical expression that indicates the ratio of the inhomogeneous surface conduction effect within the EDL to the uniform electrolyte bulk conductivity).

## 4. Conclusions

In short summary, we have provided results in terms of both mechanism analysis and numerical simulation, to account for the phenomenon of the directional-controllable unidirectional transport of a Janus mobile electrode (ME) free from buoyancy in a uniform background electric field within a broad frequency range, which is from below the inverse RC time constant for the capacitive charging of the IDL on the conducting end to even far beyond the Debye relaxation frequency of the bulk electrolyte. In stark contrast with the ICEP translation at low field frequencies, a new physical phenomenon, named “ego-dielectrophoresis (e-DEP)” is found to exist in the high frequency range, wherein the IDL has already been short circuited by a huge displacement current flowing across the Debye layer. In this way, e-DEP-enabled motion of the Janus colloid is of a pure electrokinetic origin, which behaves specifically as asymmetric interfacial charge relaxation in a homogenous electric field.

Our work justifies for the first time that a buoyancy-free Janus ME or an array of such can be transported unidirectionally in the direction of the conducting end in the high-frequency limit due to the action of driving force from e-DEP, which is right against the movement of ICEP in low frequency range. The scaling characteristic of the translating speed of e-DEP due to localized field perturbation abides by *U_e-DEP_* ∝ *V*^2^*R*, which differs from the traditional DEP velocity scale of a homogenous particle in an externally-imposed field gradient *U_DEP_* ∝ *V*^2^*R*^2^. So, in practice, it is not necessary for us to arbitrarily increase the size of the Janus ME for achieving a quicker e-DEP-enabled translating speed. Meanwhile, the phenomenon of e-DEP poses a lower demand on the electric conductivity of the liquid suspension as compared to ICEP which can only exists in dilute electrolyte.

The bidirectional moving behavior of the Janus mobile electrode in a wide frequency range is directly pertinent to the handling of nanoscale biological contents in microfluidic channels. The most salient feature of this is its robust dual-functionality in unidirectional delivery of the primary particle (Janus ME) and electroconvective collection of the secondary particles (biomolecules) on the surface of an array of the Janus mobile electrode at the same time. Future research efforts will focus on the practical experimental observation of Janus ME-based electrohydrodynamic microfluidic chips, which exceeds the scope of current analysis. It is believed that the AC electrokinetic behavior of buoyancy-free Janus mobile electrode would actively boost the interdisciplinary research on analytical chemistry, on-chip bioassay, and particle-particle electrokinetic interaction in modern microfluidic systems.

## Figures and Tables

**Table 1:** Geometric size of the fluidic device shown in Figure 1.

Symbol	Implication	Value
*H_C_*	Channel height	50 μm
*L_C_*	Channel length	300–600 μm
*W_C_*	Channel Width	500 μm
*R*	Radius of the Janus ME	3–9 μm
*H_mem_*	Gold membrane thickness	50 nm
*L_G_*	Interparticle separation	50 μm
*r*	Radius of the biomolecules	50 nm

**Table 2:** Boundary conditions for the simulation domain shown in Figure 1d implemented in Comsol Multiphysics.

Boundary Index	Electric Field Equation (2) (Ω_1_ + Ω_2_ + Ω_3_)	Flow Field Equation (12) (Ω_1_)	Solid Mechanics Equation (9) (Ω_2_ + Ω_3_)	Concentration Field Equation (15) (Ω_1_)
A-C	Zero current flux	Zero normal stress	N/A	Open boundary
A-B	Equation (7)	No slip	N/A	No flux
B-D	Zero current flux	Zero normal stress	N/A	Open boundary
C-D	Equation (7)	No slip	N/A	No flux
F-E-H	Equation (3)	Equation (14)	Equation (13)	No flux
F-I-H	Equation (4)	N/A	N/A	N/A
F-G-H	Equation (5)	Equation (14)	Equation (13)	No flux

**Table 3:** Simulation parameters pertinent to the microfluidic device taking advantage of an array of Janus mobile microelectrodes for continuous loading and collection of surrounding biomolecules for potential biomedical applications.

Symbol	Implication	Value
*σ_f_*	Liquid conductivity	0.001 (ICEP)–0.1 S/m (e-DEP)
*σ_gold_*	Membrane conductivity	10^7^ S/m
*σ_ps_*	Conductivity of polystyrene	0 S/m
*ε_f_*	Liquid permittivity	80ε_0_
*ε_gold_*	Gold permittivity	10ε_0_
*ε_ps_*	Permittivity of polystyrene	3ε_0_
*ε*_0_	Vacuum permittivity	8.85 × 10^−12^ F/m
Dion	Diffusivity of ions	2 × 10^−9^ m^2^/s
*f_RC_*	RC charging frequency	1.7–20.9 kHz
*f_MW_*	Debye relaxation frequency	225 kHz–22.5 MHz
*τ_RC_*	RC time constant	7.62 × 10^−6^–9.19 × 10^−5^ s
*τ_MW_*	Debye time constant	7.1 × 10^−9^–7.1 × 10^−7^ s
*λ_D_*	Debye screening length	3.76–37.6 nm
*C_S_*	Stern layer capacity	0.8 F/m^2^
*C_D_*	Diffuse layer capacity	0.0188–0.188 F/m^2^
*C*_0_	Initial molecule concentration	1 nM
*T*	Ambient temperature	293.15 K
*D*	Diffusivity of nanoparticles	4.3 × 10^−12^ m^2^/s
*ρ*	Liquid mass density	1000 kg/m^3^
*η*	Liquid dynamic viscosity	0.001 Pa·s
*V_1_*	Voltage amplitude	1–2 V
*f*	Signal frequency	100 Hz–500 MHz
*E*	Young modulus	10^12^ Pa
*ν*	Poisson’s ratio	0.33

## Supplementary Materials

The following are available online at https://www.mdpi.com/2072-666X/11/3/289/s1, Video S1: Rotation and translation of the Janus ME by ICEP at 100 Hz. Video S2: Rotation and translation of the Janus ME by eDEP at 10 MHz. Video S3: Loading of SP by the mobile PP driven by ICEO streaming at 100 Hz. Video S4: Loading of SP by the mobile PP driven by eDEP-enabled flow at 10 MHz. Video S5: Biomolecule concentration using an array of Janus mobile electrode.
